# Students’ Perceptions of Team-based Learning in an Undergraduate Nutrition School

**DOI:** 10.15694/mep.2018.0000226.1

**Published:** 2018-10-02

**Authors:** Patricia Constante Jaime, Cláudia Raulino Tramontt, Kamila Tiemann Gabe, Lígia Cardoso dos Reis, Tarsis de Mattos Maia

**Affiliations:** 1 School of Public Health /University of São Paulo

**Keywords:** teaching and learning, public health, communication skills, undergraduate

## Abstract

This article was migrated. The article was marked as recommended.

**Introduction:** Current challenges in the food and nutrition fields have required training nutritionists to develop teamwork skills, demanding the use of active methodologies.

**Methods:** This qualitative descriptive study aimed at knowing Nutrition students’ perceptions about the use of Team Based Learning (TBL) in a course. Focus groups were conducted and submitted to content analysis.

**Results:** Categories were grouped into three axes: (1) principles of the method ( *teamwork*, *individual preparation* and *teacher’s role*), (2) results of the experience ( *knowledge acquisition*, *critical judgment* and *communication/argumentation*) and (3) meanings of the experience ( *engagement/motivation* and *preference for the method*). *Teamwork* was the most frequent category (51 occurrences), followed by *knowledge acquisition* (44) and *engagement/motivation* (35). Axis 1 categories came out linked to each other and to at least one Axis 2 category, revealing the course’s coherence with TBL principles, thus allowing better knowledge acquisition, communication skills, and development of critical judgment. Axis 3 was connected to the others, pointing out that both principles of the method and results of the experience contributed to students’ engagement and preference for the method.

**Conclusion:** According tostudents’ perceptions, TBL can contribute to provide nutritionists with better technical training, critical judgment, and communication skills.

## Introduction

The field of Food and Nutrition has gained prominence in the global agenda and under the Sustainable Development Goals as a result of the commitment signed by several countries, within the *United Nations Decade* of *Action* on *Nutrition* (2016-2025), to eradicate hunger and all forms of malnutrition ( FAO and WHO, 2016). Facing these challenges demands a highly qualified Nutrition workforce with technical-scientific knowledge as well as enhanced communication, management, teamwork and problem-solving skills ( Fanzo *et al*., 2015). Gaps in nutritionists’ training have been pointed out ( Recine and Mortoza, 2013) and indicate the need to review pedagogical projects to ensure humanistic, generalist and critical performance as well as decision-making, communication and leadership skills ( Alves and Martinez, 2016).

A major challenge posed to undergraduate Nutrition Schools is the use of teaching-learning active methodologies that focus on problematization ( Recine and Mortoza, 2013). They must lead to the development of key skills for future professional activity such as teamwork as well as political and management leadership ( Frenk *et al*., 2010). In response to this context, the use of Team Based Learning (TBL) to train health professionals has increased in recent decades in order to encourage critical thinking and change the education system in the ( Reimschisel *et al*., 2017). Nevertheless, studies evaluating the use of this methodology in undergraduate Nutrition Schools have not been found so far.

TBL is an educational strategy in which students are organized in multiple and small groups (5-7 individuals) to discuss contextualized problems in scenarios prepared by teachers. These discussions are designed to occur with and among teams, encouraging reasoning and debate ( Haidet *et al*., 2012). Application of TBL requires a sequence of events aimed at maximizing learners’ preparation and participation, namely: **Step 1** - Preclass preparation (individual study); **Step 2** - Ensuring student’s readiness (Readiness Assurance Test, RAT) applied individually (iRAT) and in groups (gRAT), followed by written team appeals and teacher feedback; **Step 3** - Application of concepts through group problem-solving activities ( Haidet *et al*., 2012).

TBL’s theoretical framework is grounded on constructivism, since the teacher becomes a facilitator of learning in an environment free of authoritarianism, takes into account students’ previous experiences and knowledge, uses dialogue and interaction as a means for acquiring knowledge, and encourages students’ thinking and reasoning in and about practice ( Hrynchak and Batty, 2012). Considering the lack of publications in this context, the present study analyzed perceptions of Undergraduate Nutrition students on the use of TBL in the course *Public Policies on Food and Nutrition (PPF&N).*

## Methods

This is a qualitative descriptive study to discuss the first application of TBL in *Public Policies on Food and Nutrition* (PPF&N) in two classes of the 4 ^th^ year of an undergraduate Nutrition School. PPF&N is mandatory and it is part of the Nutrition School program. It aims at knowing and analyzing Food and Nutrition Public Policies in face of Brazil’s epidemiological, social and political scenarios.

### TBL in the PPF&N Course

Fifteen 4-hour meetings (60 hours) were held. In the first meeting, the teacher presented TBL steps, the teaching plan, the schedule of activities, and the random division of teams (5-6 members). The other meetings were conducted as follows: one for a lecture and 13 for TBL sessions.

For Step 1, one week before each meeting, preparatory material was provided online (theoretical synthesis of the content; a situation based on simulated scenario developed by the teacher; and complementary bibliography). In Step 2, conducted in classroom, students took the Readiness Assurance Test (RAT), individually at first (iRAT) and then in teams (gRAT). It included 7-8 multiple choice questions based on the material provided, which could not be consulted during the test. At the end of each session, the teacher provided feedback through a lecture. Teams could appeal missed questions to the teacher within 72 hours (The University of British Columbia, n.d.). Step 3 was based on a problem situation in which teams should draw up a Food and Nutrition intervention plan. Students were evaluated according to their performances in this step and in the tests (iRAT and gRAT), in addition to peer evaluation within teams.

### Data Collection and Analysis

In order to understand students’ perception of the experience, a focal group (FG) was used for data collection. At the end of the course, each class was divided into two groups (11-17 students) for the FG conducted by an experienced mediator in the presence of an observer. Topics covered included: (1) expectations toward the TBL method on the first day of class; (2) skills and competences developed at each TBL step; (3) the teacher’s role in TBL; (4) the teaching-learning process; (5) challenges experienced during the course with the new method. FG meetings were recorded in audio and fully transcribed.

The data collected and transcribed were submitted to content analysis ( Ryan and Bernard, 2010). The categories were established a posteriori using an inductive approach and codebooks were produced ( Ryan and Bernard, 2010), which were applied by two pairs of coders. Inter-rater reliability was analyzed by agreement between pairs with Kappa coefficient (considered good when ≥0.6) calculated with GraphPad QuickCalcs (QuickCalcs. 2017 GraphPad Software Inc. La Jolla, CA, USA). A fifth coder (the main researcher) defined consensuses. Final categories and Kappa values were: *teamwork* (0.739); *teacher’s role* (0.809); *individual preparation* (0.853); *knowledge acquisition* (0.731); *critical judgment* (0.952); *communication/argumentation* (0.693); *engagement/motivation* (0.759); and *preference for the method* (0.701).

The project was approved by the Ethics Committee for Research with Human Beings of the School of Public Health/University of São Paulo (FSP/USP). All participants signed informed consent forms.

## Results/Analysis

Table 1 presents the categories grouped into three axes according to their meanings in the context studied.

**Table 1.** Axes, categories and respective inclusion criteria

Axis	Categories	Inclusion criteria
1) Principles of the method	Teamwork	Its presence was perceived in statements about learning to deal with differences; the effort to make it viable; discussions to reach consensus; commitment to classmates’ learning. Its absence, in turn, was perceived in statements about one of the members being overloaded with work; division of tasks for individual work; or decisions made by voting rather than consensus within the team.
	Individual preparation	Perception about organization of the weekly study routine and good performance in tests (iRAT and gRAT); commitment to team discussion; or, on the other hand, explicit indication that there was no individual preparation.
	Teacher’s role	Perception of the teacher as a facilitator of learning who establishes a horizontal relationship with learners or just transfers knowledge (inconsistent with the method).
2) Results of the experience	Knowledge acquisition	Perception that the method enabled knowledge acquisition; its steps were essential for learning and there was contextualization of content in professional practice.
	Critical Judgment	Expression that the method encouraged questioning and analysis of situations experienced and the information received.
	Communication/argumentation	Demonstration that the method provided improved communication skills and increased self-confidence to discuss/argue.
3) Meanings of the experience	Engagement and motivation	A feeling of encouragement to performing tasks and participating in every step of the method; arousal of affinity with/interest in the content taught; encouragement to get involved with the course through perception of the teacher’s dedication.
	Preference for the method	Explicit preference for TBL over other methods used in the course.

*Teamwork* was the most frequent category (51 occurrences), followed by *knowledge acquisition* (44) and *engagement/motivation* (35). Figure 2 illustrates the occurrence of all categories that emerged, whether they appeared disconnected and interconnected. The sizes of the circles are proportional to each category’s frequency and the magnitude of intersections between them. Axis 1 categories (principles of the method) appeared linked to each other and to at least one Axis 2 category (results of the experience), thus forming three pairs: “ *teamwork/communicationand argumentation*”, “ *teacher’s role/critical judgment*” and “ *individual preparation/knowledge acquisition*”. As a whole, Axis 1 was linked to *engagement and motivation* (meanings of the experience), which, in turn, was also linked to the other category belonging to the same axis: *preference for the method.*

**Figure 1.**
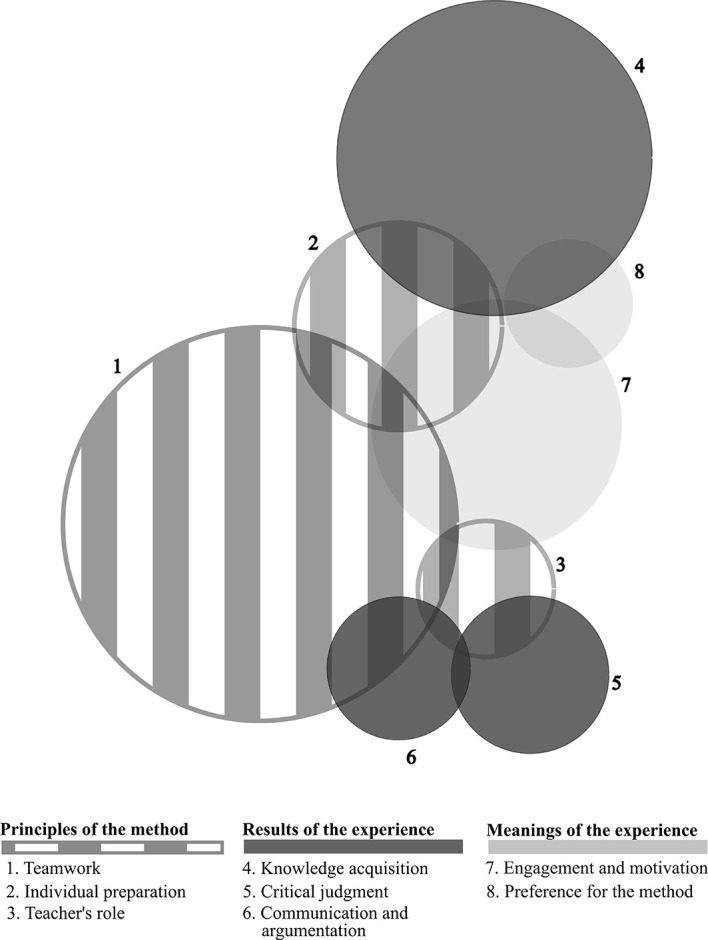
Frequency and intersection of categories

### 1) Principles of the Method

The three categories that emerged in this axis revealed the coherence of TBL’s principles with the work developed during the course. In addition, the categories indicated mostly good acceptance and approval of the methodology by the students.

The category *teamwork* was often linked to *communication/argumentation*, followed by *individual preparation* and *engagement/motivation.* There was concern with individual preparation to contribute to teamwork during the discussion of the questions in classroom. This form of work was said to inhibit mere division of tasks and encouraged their shared and committed execution, since the pairs, as expressed in statements, would not tolerate lack of commitment. On the other hand, some teams had problems with lack of commitment by one or more members, which hampered discussions and overloaded their colleagues with work.

*Individual preparation* was more often associated with *engagement/motivation*, followed by *knowledge acquisition* and *teamwork*, and perceived mostly as a positive aspect of the course. Such relationship was noticed when students’ statements indicated that this principle led them to create their own study routines, showing responsibility, which, in turn, was related to concern with their performance in tests and their commitment to the team. Another aspect facilitating individual preparation was the characteristics of the texts provided: they were short, objective, theoretically well-grounded and didactic. Individual preparation, when absent, was said to hamper teamwork and was described as “ *lack of responsibility*”.

*Teacher’s role* was associated with critical judgment. Students identified the teacher as the mediator of the learning process, who encouraged knowledge construction and autonomous search for information. The horizontal relationship between educator and learners was evidenced by the perception of a learning environment open to questions, exchange of knowledge between learner and educator, participation, and encouragement to use critical judgment. On the other hand, the teacher’s role was perceived differently at the feedback step, similar to lectures typical in more traditional/conventional methodologies.

### 2) Results of the experience

In this axis, categories emerged linked to the previous ones, all suggesting that the course allowed better knowledge acquisition and developed communication/argumentation and critical judgment skills.

The category *knowledge acquisition* varied from understanding the content itself from a cognitive perspective to perception of the development of skills needed to apply that content in professional practice. It was mainly related to *individual preparation* seen as the *trigger* for learning. The use of a simulated scenario seemed to hold potential for contributing both to knowledge acquisition and its future application in real-life professional situations.

Three elements were perceived as necessary for developing *communication/argumentation*: 1) an environment open to discussion, favoring communication, provided mainly by teamwork and the horizontal relationship established by the teacher; 2) a theoretical knowledge basis for arguing, having studied the material provided; and 3) knowing how to articulate and present the arguments - skills related to the environment and the theoretical knowledge basis.

Development of *critical judgment* was linked to greater opening to questioning and debate with the teacher (horizontal relationship). This relationship encouraged a questioning and challenging attitude and the search for answers (information). The appeal was considered the TBL step that most aroused critical judgment by allowing students to improve their argumentation skills. In addition, *critical judgment* was also associated with *individual preparation*, which strengthened their arguments and elicited reflections on public policies, nutritionists’ role in the area, and the population’s rights.

### 3) Meanings of the Experience

This axis was related to both principles of the method and results of the experience. Factors that contributed to *engagement and motivation* included the need for continuous study, shown in student’s statement on both *individual preparation* and the tests; commitment to *teamwork*; and *teacher’s role* - where workload and the dedication necessary to use TBL were mentioned as well as the horizontal relationship established with the students throughout the course. *Preference for TBL* over conventional methods, in turn, was related to the perception that they were able to learn - to acquire knowledge - better. Dynamism of the method was pointed out as determinant for *engagement/motivation* and *preference for TBL.*

The statements that best represent each category are presented in Table 2.

**Table 2.** Examples of representative statements for each category

Axis	Categories	Examples
Principles of the method	Teacher’s role	“I think she is a mediator. She won’t show that she’s got all the knowledge. She provided us with knowledge, showed us where it is, where we can find it. She gave us some bibliography and everything, but in the end, which was the application of TBL, we had to look for it, we went after it and everything”.
	Teamwork	“I felt that these group discussions... they acted like there was integration of the team’s members’ thoughts, like a clear view focusing... I don’t know, on the patient... the population or the target of that policy, but in a way, like, a conversation between professionals, you know?”
	Individual preparation	“TBL made no sense if you hadn’t’ read the synthesis, you wouldn’t have anything to discuss with the group, you wouldn’t be able to answer the questions. So, if you hadn’t read it, at least read it, it made no sense, the method was meaningless. So I think the main point of learning was reading”.
Meanings of the experience	Engagement and motivation	“Actually, when I saw the name of the course early in the semester I said ‘wow, this course will really make me sleep’. I had huge prejudice in the beginning. [...] To meet other people, to work with them, it was a great challenge, it was really cool, [...] You felt motivated and you’d say: ‘Gosh, I want to know everything so I can answer it right, I want to do well in this’, and it was really cool and I was very open to this course. It instigated us to want to know more about it and how important our role in that area is”.
	Preference for the method	“If it had been taught in a traditional format, like slides or passing a test, it would’ve gone unnoticed, but the way it was, at least for me, even if it’s not something I particularly like [the PPF&N area], I enjoyed the course. I’m more open to it today than I was at the beginning”.
Results of the experience	Knowledge acquisition	“I think it showed how we do public policy in practice; it’s the actual policy there, with the manager, not just on paper... It’s policy in the relationship, in the way of talking and in what to do and how to do it. So, I think it was public policy really experienced, not just on paper”.
	Communication/argumentation	“I think the interesting part was that you had to justify and argue for your answer, then you could not just answer and say: ‘Ah, I answered A, can’t it be A?’. No, people answered different things and each one defended their point of view and people said: ‘Oh, I guess yours is not right because of this and that’, and you can debate... I think in our group, at least, the whole thing was better than I expected”.
	Critical judgment	“... I think it was an interesting exercise and I’m grateful to her for this possibility, and I repeat, it’s a shame that it happened only in the last semester [of Nutrition School], right? It would’ve been good if it had happened before, right? And maybe it would encourage this kind of feeling in students, of really seeking answers, right? ... And not conforming, that’s very important in life, right? We disagree with things, and even more in the current situation, I guess ... So ...”.

## Discussion

The course was designed using a TBL approach to foster knowledge acquisition in PPF&N and development of teamwork skills, making students active in the learning construction process, with greater critical awareness about context and reality. Teaching public policies involves an intrinsic commitment to train professionals who are able to deal with multifactor situations/problems that require multidisciplinary/interdisciplinary and often intersectoral work ( Frenk *et al*., 2010; Recine and Mortoza, 2013).

The TBL method seeks to fill this gap in health professionals training, being a powerful methodology to develop teamwork, communication, problem-solving and conflict mediation skills for students who experience it. These potentialities justify the method’s choice to restructure the course’s methodology. Studies evaluating the effect of TBL on the development of these competencies and skills in health higher education have also shown positive results ( Frame *et al*,. 2015; Huggins and Stamatel, 2015; Bleske *et al*., 2016; Reimschisel *et al*., 2017; Remington *et al*., 2017; Zeng *et al*. 2017).

The use of teamwork as a learning tool is seen as reinforcing possibilities for participation and interaction among students ( Reimschisel *et al*., 2017). This relationship makes sense if we note that teamwork was perceived when participation in discussions was uniform, with good synchrony and adaptations to differences in ways of thinking and working, always under a respectful relationship.

In this context, the time for discussions is an opportunity to exercise and improve communication skills ( Remington *et al*., 2017). TBL is known to be effective in enabling health professionals to work collaboratively while exercising their communication skills with improved decision-making, negotiation, and respect for colleagues ( Hrynchak and Batty, 2012; Currey *et al*., 2017; Reimschisel *et al*., 2017).

Relations perceived between *teamwork* and the categories *individual preparation* and *engagement/motivation*, as well as *communication/argumentation*, can be explained by dynamics inherent to the method. As learners act in teams, they tend to study before classes, making discussions more dynamic and increasing their confidence to argue among themselves and with the teacher. This dynamic seems to favor friendly coexistence with colleagues and the teacher, making the environment more pleasant and motivating.

On the other hand, in this study, the absence of teamwork was noticed when at least one group member did not commit to the activities, hampering discussions and overloading colleagues with tasks. According to Frame *et al*. (2015), students often resist implementation of TBL or any other active method because they have to change the passive attitude common to the lecture-format classes. In addition, the authors see conflicts as inevitable when working in groups, which may impact students’ perceptions about TBL.

Regarding *individual preparation*, Remington *et al*. (2017) pointed out that this principle also contributed strongly to learning, similarly to the findings of this study. Evidence indicates that students tend to study 2-3 times longer under TBL when compared to traditional methods ( Zeng *et al*., 2017). Perception of *individual preparation* as a crucial stage for learning, noted in this research, seemed to be related to the characteristics of the texts provided, which facilitated reading and organization of the study routine. In turn, Zeng *et al*. (2017), pointed at the tests as the core factor for students’ commitment to self-learning.

It is important to emphasize that TBL requires that both learners and educators fulfill its steps and that the latter change their relationship with the students ( Gullo, Ha and Cook, 2015). In this context, the teacher was recognized as mediator of the autonomous learning process, encouraging knowledge construction and critical judgment. Studies have shown that the tests and tasks performed in TBL allow exercising critical thinking by putting the knowledge acquired into practice in a complex scenario ( Frame et al., 2015; Gullo, Ha and Cook, 2015; Huggins and Stamatel, 2015; Bleske et al., 2016). Critical thinking is also achieved through discussions with teammates, favoring joint learning ( Gullo, Ha and Cook, 2015). In this study, the horizontal relationship established between teacher and students triggered critical thinking already encouraged by the TBL method.

However, the moment when the teacher takes center stage - the feedback step - was perceived as a lecture-format class by students. This contrast to TBL’s methodological characteristics seems to have contributed for students to underestimate the importance of that step for learning.

The “ *meanings of experience*” axis bears elements of others (“ *principles of the method*” and “ *results of the experience*”), pointing to students’ sense of engagement/motivation and preference for the method. Haidet *et al*. (2012) created a conceptual model where engagement is the core element, defining it as students’ involvement with content and teamwork. In that model, teachers’ decisions are used as mediators for students’ engagement, which in turn will determine academic success in acquiring knowledge and developing teamwork, communication and leadership skills. The results found in this study are in line with that perspective, since they suggest that the principles of the method were the main factors to determine engagement/motivation toward classes. In this study, the teacher’s dedication - perceived in relation to the workload demanded by the method - and the horizontal relationship established with students worked as motivational determinants.

Students’ preference for the teaching method is recognized as an important element to motivate learning ( Mangold, 2007). In this study, it was attributed to higher knowledge acquisition. Several statements by students indicate that TBL could be used in other Nutrition School courses as a teaching strategy appropriate for professional practice.

The systematic review conducted by Reimschisel *et al*. (2017) revealed lack of studies on the impact of TBL in the Nutrition field in developing countries. It included 118 TBL studies in undergraduate Health Schools, most of them related to Medicine and Pharmacy, and none in Nutrition Schools. However, it is well known that nutritionists must be prepared beyond the “ *assistencialism*” perspective, learning to work in teams and perform leadership and management functions under an interprofessional perspective, given the challenges posed by today’s global epidemiological scenario ( Recine and Mortoza, 2013). Therefore, a strength of this study lies in its originality and pertinence when applying and evaluating the use of TBL in a course of an undergraduate Nutrition School.

Limitations described in studies using TBL include low number of sessions (five on average) and evaluation of the method exclusively through questionnaires that generate scores ( Reimschisel *et al*., 2017). This study investigated a course taught almost entirely through TBL sessions, employing a qualitative approach that considers students’ subjective perceptions.

However, voluntary participation in FGs might have caused bias as a result of inclusion of students who were more engaged with and motivated by the method, amplifying positive perceptions. Another possible limitation of this study was random division of teams. The literature indicates that TBL participants should be chosen to enable a diverse and balanced team composition ( Parmelee and Michaelsen, 2010; Parmelee *et al*., 2012). While randomization was employed, chance errors might have happened, which would leave students with similar characteristics on the same team.

## Conclusion

According to students’ perceptions, the three basic TBL principles - *teamwork*, *individual preparation for classes*, and the *teacher’s role* as a facilitator of learning - were recognized in the PPF&N course. They reported increased *knowledge acquisition*, improved *communication/argumentation* skills, and development of *critical judgment.* Both the enhancement of these skills and TBL principles contributed to higher *engagement/motivation* toward the course and *preference* for that learning method. Therefore, the results of this study suggest that the use of TBL in Nutrition instruction can contribute to train nutritionists with better technical knowledge, critical judgment, and communication skills.

## Take Home Messages

• TBL has been widely used in health education, but no studies were found addressing higher education in Nutrition.
• TBL was considered effective for enhancing knowledge, communication/argumentation and critical judgment skills.
• TBL was considered suitable for the learning process and for developing important professional skills.
• Individual preparation for classes seems to have placed students in some sort of cycle: reading the texts increased their knowledge, which in turn triggered motivation to study more.
• Engagement/motivation toward the course and preference for TBL were associated with its principles and with the development of the skills mentioned.

## Notes On Contributors

PCJ is an Associate Professor at The School of Public Health/University of São Paulo (USP) and a Researcher at the Center for Epidemiological Research in Nutrition and Health (NUPENS/USP).

CRT is a PHD student on the Nutrition in Public Health Graduate Program at the School of Public Health/USP.

KTG is a master’s degree student on the Nutrition in Public Health Graduate Program at the School of Public Health/USP.

LCR is a PHD student on the Nutrition in Public Health Graduate Program at the School of Public Health/USP.

TMM is a PHD student on the Nutrition in Public Health Graduate Program at the School of Public Health/USP.
