# Supplementary material for: Students’ Perceptions of Team-based Learning in an Undergraduate Nutrition School
Source: MedEdPublish (2016). 2019 Jul 16;7:226. Originally published 2018 Oct 2. [Version 2] doi: 10.15694/mep.2018.0000226.2 (PMC10917459; doi:10.15694/mep.2018.0000226.2)
Supplement: Peer review history

# Reviewer report: Reviewer response for version 2

**DOI:** 10.21956/mep.20233.r31351

**Published:** 2019-08-04

**Authors:** Tharin Phenwan

**Affiliations:** 1 University of Dundee

This review has been migrated. The reviewer awarded 3 stars out of 5 First of all, I must commend the authors to improve their work and it is even better than the first version.The article is much better and easier to navigate through. Overall, this is a good article that will be of interest to those who are involved in teaching and curriculum planning.

Reviewer Expertise:

NA

No decision status is available

# Reviewer report: Reviewer response for version 2

**DOI:** 10.21956/mep.20233.r31354

**Published:** 2019-07-29

**Authors:** Augusto Scalabrini Neto

**Affiliations:** 1 University of Sao Paulo School of Medicine

This review has been migrated. The reviewer awarded 2 stars out of 5 This is mostly a reaction study to TBL using Nutrition students. The method has been widely discussed in other studies, and it does not seem that a new knowledge emerged from this description.

Reviewer Expertise:

NA

No decision status is available

# Reviewer report: Reviewer response for version 2

**DOI:** 10.21956/mep.20233.r31352

**Published:** 2019-07-27

**Authors:** sathyanarayanan varadarajan

**Affiliations:** 1 SRM Medical College, SRM University

This review has been migrated. The reviewer awarded 4 stars out of 5 This is a good article based on the qualitative descriptive study which has analyzed perceptions of Undergraduate Nutrition students on the use of Team Based Learning (TBL) in the course Public Policies on Food and Nutrition (PPF&N). In order to understand students’ perception of the experience, focal groups (FG) were used for data collection. The results of this study suggest that the use of TBL in Nutrition instruction can contribute to train nutritionists with better technical knowledge, critical judgment, and communication skills. Though this study has some limitations, it has contributed well to the field of higher education in Nutrition where no studies were found addressing this issue. It will be helpful to medical educators who are involved in curriculum planning and development for higher education in Nutrition to consider TBL as an effective educational strategy for enhancing knowledge, communication/argumentation and critical judgment skills.

Reviewer Expertise:

NA

No decision status is available

# Reviewer report: Reviewer response for version 2

**DOI:** 10.21956/mep.20233.r31353

**Published:** 2019-07-16

**Authors:** Ken Masters

**Affiliations:** 1 Sultan Qaboos University

This review has been migrated. The reviewer awarded 4 stars out of 5 This is a revised version of the previously-submitted paper. I am pleased to see that the authors have used the opportunity to address the issues raised by the reviewers of the original paper.One area, however, that could still have had more information as requested, was on the running of the focus groups. Although a little more information has been given about the these, not all the requested information has been supplied. In particular, the length of time that the focus groups were run, the numbers and types of questions given to the participants, etc. (Although the script structure is given, more details would have helped – compare this to a quantitative survey, in which supplying a copy of the survey form is relatively standard.).Nevertheless, a useful improvement.

Reviewer Expertise:

NA

No decision status is available

# Reviewer report: Reviewer response for version 1

**DOI:** 10.21956/mep.19116.r27580

**Published:** 2018-10-11

**Authors:** Tharin Phenwan

**Affiliations:** 1 University of Dundee

This review has been migrated. The reviewer awarded 3 stars out of 5 An interesting paper to assess the undergraduate students’ perception regarding TBL.Good details on the course and rationale to conduct the research with supporting reference.-The authors described the participants to some degree and the overall usage of the focus group and content analysis but do not give deeper details of how they collected and analysed the data. Rigour was also not mentioned; how do they increase the rigour of this work? How many researchers analyse the data and how did they deal with discrepancies? How did they develop the codebook? - They did not explain the context clearly. How does the curriculum look like? How many students were there? Were they all recruited?- Some remark on the ethical aspect of this classroom research. Will the students feel coerced to join as participants since they were obliged to join this course as parts of their learning? And how did the authors deal with this situation?-On the methodology section, how long did the interviews last? How did the authors create the codebook? Were there any field notes or audit trails during the process? Participant demographical data was also not mentioned. -Results section could be improved. I believe that the readers still cannot picture the participants. What are they like? Is it one gender-dominated? What about their grades? Were there any specific differences or similarity between the groups? -Table 1 is comprehensive, but the inclusion column could be easier to navigate. This also applies to table 2. The examples column is rather hard to follow through. It may be easier to read if the authors selectively pick up the most meaningful quotes. - Figure 1 captures the good summary of the texts, making it easier to follow. There is also a minor correction to change the word "figure 2" to "figure 1" in the main text. -Implications for future works were not clearly mentioned. How will you continue to monitor these participants. And how will you assess them more objectively in the future? Overall a good article that will be an interest of those who are involved in teaching and curriculum planning.

Reviewer Expertise:

NA

No decision status is available

# Reviewer report: Reviewer response for version 1

**DOI:** 10.21956/mep.19116.r27579

**Published:** 2018-10-08

**Authors:** Ramon Esperon-Hernandez

**Affiliations:** 1 University of Yucatan

This review has been migrated. The reviewer awarded 3 stars out of 5 The idea of the article is good and contributes the experience gained in the implementation of the Team Based Learning (TBL) like pedagogical strategy in the course Public Policies on Food and Nutrition.The methodology is not clear regarding the data collection and qualitative analysis, I suggest to describe it better. The organization of the focus groups is confusing; it is difficult to understand that there were four focus groups of 11-17 subjects. It is assumed that being four focus groups required two pairs of coders, a total of four. It does not specify if you used any software for qualitative analysis.Figure 1 is referred to in the text as figure 2. It is very illustrative, I suggest mentioning the name of the representation technique used to elaborate figure 1. I suggest inserting labels to each circle with the name of the category; it is difficult to make an analysis with the numbers.About the conclusions, although the TBL strategy has been accepted in the literature as valuable, and the perceptions of the students are good, I consider that the design of the study does not allow affirming the increase or improvement of the skills, because don’t were compared before and after the course.

Reviewer Expertise:

NA

No decision status is available

# Reviewer report: Reviewer response for version 1

**DOI:** 10.21956/mep.19116.r27581

**Published:** 2018-10-06

**Authors:** Barbara Jennings

**Affiliations:** 1 Norwich Medical School

This review has been migrated. The reviewer awarded 4 stars out of 5 This article by Patricia Constante Jaime et al about TBL resonated with me because of my roles as a tutor for problem based learning (PBL), and curriculum planner. I enjoyed reading the paper and thinking about the various approaches that are possible in small group settings, using a constructivist approach. I will share the article with colleagues, along with some key papers cited by the authors that explore the theoretical framework and effectiveness of TBL.This paper is well-written, with strengths in the literature review; the design of the associated study; and in its description of the evidence-based TBL intervention (which reads like an interesting case report in itself) in the School of Public Health at the University of Sao Paolo.The content analysis was clearly described and justified and the results were clearly presented and discussed. Figure 1 is a really informative graphic which gives an immediate overview of the weighting and intersection of the range of student’s perceptions.The role of the “teacher” is (quite rightly) not perceived to be a major issue by the students. The educators’ collective responsibilities are to develop the right learning environment and educational materials to promote stepwise active-learning and teamwork, so much of it this is behind the scenes and light-touch facilitation. The authors discuss the transition that occurs when the horizontal student-teacher relationship morphs into a more didactic lecturer-student interaction for the feedback step.Although the acceptability and effectiveness of TBL are well-established in the literature, the novelty of this study is in its application to a nutrition and public policy setting.

Reviewer Expertise:

NA

No decision status is available

# Reviewer report: Reviewer response for version 1

**DOI:** 10.21956/mep.19116.r27578

**Published:** 2018-10-06

**Authors:** Ken Masters

**Affiliations:** 1 Sultan Qaboos University

This review has been migrated. The reviewer awarded 3 stars out of 5 Overall, a useful study. A little more detail replied, and some small changes in the presentation of the data would be appreciated.I would like to see a more information about the numbers of students. The number of students on the course does not appear to be given. With the focus groups, I see only that “each class was divided into two groups (11-17 students) for the FG conducted by an experienced mediator in the presence of an observer.” This would tell me that each class was between 22 and 34 students, but that is very vague. So, I would like to see specifics of the class size and of each focus group (and I’m assuming there were only two focus groups, but the paper speaks of the FG in the singular). I would also like to see a little more description about the FG process followed. (Time, number and types of questions, etc.). “approach and codebooks were produced” – Are these codebooks books of codes? This needs a little more explanation.I can understand the logic of combining the examples into Table 2, but, from a reader’s perspective, it would be better if the examples could be given in their respective section. This is in keeping with what readers expect, and is easier to understand, so that readers do not have to return to the previous section to try to match examples to content. A few more examples would also be nice – you have the space, so please use it. (If you don’t wish to break the flow of the paper, then please supply them in supplementary files).I would also like to see some discussion about how generalisable the authors believe these results to be.

Reviewer Expertise:

NA

No decision status is available
